# Combining tangential hydrodissection, panniculectomy, and negative pressure wound therapy in treating extensive degloving injury of the leg

**Published:** 2014

**Authors:** E Albu, A Alexandru, B Marinescu, R Ene, C Cârstoiu

**Affiliations:** * ”Carol Davila” University of Medicine and Pharmacy; Clinic of Plastic Surgery and Reconstructive Microsurgery, University Emergency Hospital, Bucharest, Romania; ** “Carol Davila” University of Medicine and Pharmacy; Clinic of Plastic Surgery and Reconstructive Microsurgery, Central Military University Emergency Hospital, Bucharest, Romania; *** “Carol Davila” University of Medicine and Pharmacy; Department of Orthopaedics and Traumatology, University Emergency Hospital, Bucharest, Romania

**Keywords:** degloving injury, Versajet, full-thickness skin graft, negative pressure wound therapy

## Abstract

Major degloving injuries of the lower limb are daunting lesions because they are relatively rare and always produce larger soft tissue defects than direct visual inspection that could be predicted in the emergency room. Enough body of the medical literature supports the excision of the avulsed tissue and replaces it as a full-thickness skin graft. However, almost paradoxically, there is little support for the use of pristine large full-thickness skin graft in the treatment of these lesions.

This article focuses on the use of tangential hydrodissection (VERSAJET Hydrosurgery System, Smith & Nephew) in preparing the avulsed wound and defatting of a large piece of full thickness skin graft taken from the abdomen by using a standard panniculectomy excision pattern and securing survival and integration of the graft with negative pressure wound therapy. The patient, a 60-year-old obese and diabetic woman, suffered a roadside accident producing a degloving injury of her leg and was treated with the presented method; the final result was excellent and the reduction of the abdominal panniculus was a bonus.

## Introduction

Major degloving injuries of the lower limb are high-energy trauma produced by abrasion, torsion and compression [**1**]. Depending on the duration and intensity of each of the offending forces, the resulting injury consists of an open abraded wound of variable depth and surface [**2**], an area of undermining of the soft-tissue envelope affecting a part or the full circumference of the limb in a single or multiple plane [**1**] and variable degrees of muscle crushing with or without bone fractures. The hallmark of this type of lesion is the avulsion of the septal and muscular perforator, which devascularises an important part of the skin envelope. The typical patient is a pedestrian run over by a heavy vehicle like a bus or a truck. The general rules of acute wound debridement applies, meaning that every bit of a tissue that is physically disorganized or grossly impregnated with dirt or debris should be liberally excised and the wound should be copiously irrigated. After these maneuvers are successfully performed, the surgeon is still left with one difficult decision regarding the degloved skin-envelope, i.e. to estimate which part of this flap will survive as a randomly perfuse flap, and hence leave it alone, and which part is going to die, and try to rescue it by defatting and reapplying it as a full thickness graft, immediately or in a delayed fashion [**3**]. Because visual inspection is very unreliable in judging the viability of the degloved flaps and intra-venous flouroscein, it may over-estimate the line of demarcation between viable and non-viable skin [**4**], the “wait and see” policy could be a safe decision. As soon as a visible line of demarcation is clear, the surgeon should proceed to reconstructive steps, aimed at obtaining the best possible skin graft for the best possible wound bed. This article concentrates on achieving these goals through incorporating the technique of tangential hydrodissection in preparing the wound and harvesting a large piece of full thickness skin graft from the abdomen and securing the survival and integration of the graft with negative pressure wound therapy.

## Case presentation

**Fig. 1:**
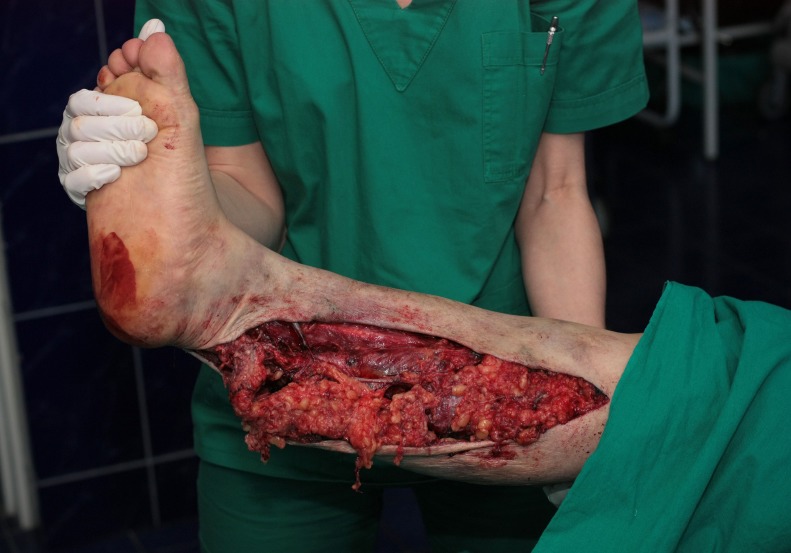
The case at presentation

A 60-year-old woman suffered a roadside accident. Her right leg was run over by one of the track tires and sustained a non-circumferential multi-planar degloving injury (**Fig. 1**); her left foot was abraded below the medial malleolus.

**Fig. 2:**
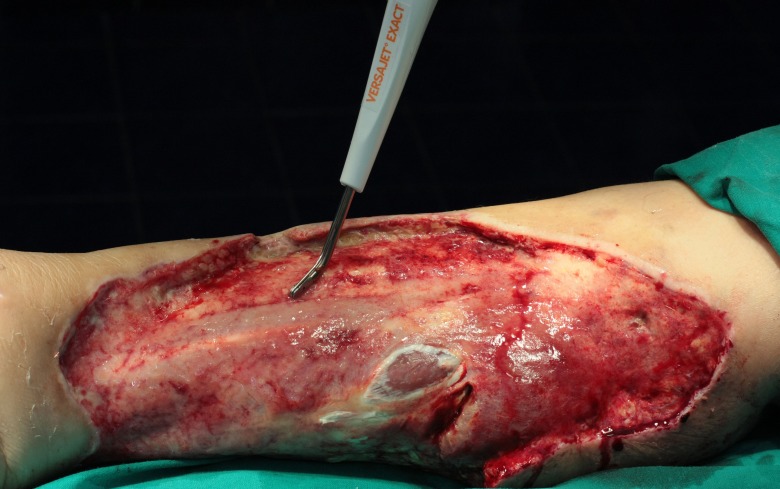
Intraoperative view of the wound being prepared with VERSAJET Hydrosurgery System

 She was admitted and underwent debridement of devitalized tissues, but because a clear hypo-perfused area of the skin could not be documented, the leg wound was thoroughly washed, sutured and drained. The patient was obese and diabetic, so the delayed full-thickness necrosis of skin and subcutaneous tissue affected almost the full length of the medial, posterior and lateral areas of the right leg, fortunately spearing a narrow bridge of skin in front of the tibia. After one week, the necrotic tissue was surgically removed and the wound responded favorably to daily changes of dressing under intravenous penicillin and gentamicin. Two weeks later the recipient bed was clean, covered with early granulation and no clinical sign of residual necrosis or infection. The final debridement was achieved with a method commonly referred to as tangential hydrodissection, by using the VERSAJET Hydrosurgery System (Smith & Nephew) connected to a 45°/8 mm hand piece and power settings of 5 to 7 (**Fig. 2**). 

**Fig. 3:**
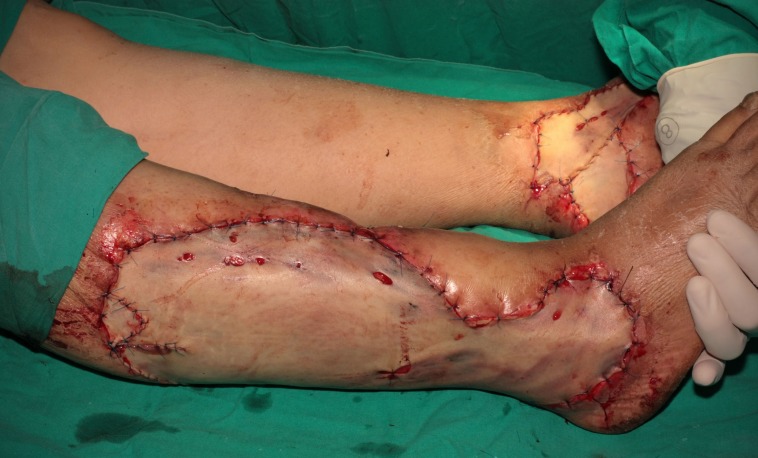
A full-thickness single piece skin graft harvested from the lower abdomen was defatted with VERSAJET and sutured in place

In the same surgical procedure, a full-thickness single piece skin graft was harvested from the lower abdomen by using a standard panniculectomy excision pattern and the same Hydrosurgery System was used for defatting the graft. The abdominal donor site was directly closed with a minimal undermining and without an umbilical transposition. The skin graft was tailored to the defects and sutured in place under mild tension with very few stab incisions to allow fluid passage (**Fig. 3**). 

**Fig. 4:**
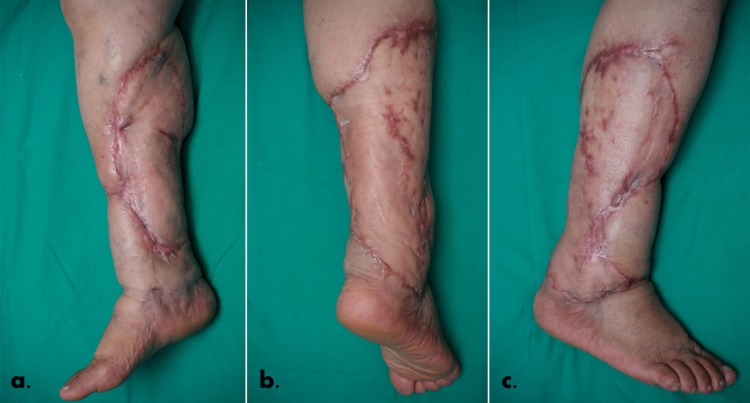
Appearance of the right leg 5 months after the accident

A gauze-based negative pressure wound therapy (NPWT) system (Renasys EZ Plus, Smith & Nephew) at 90 mm Hg continuous pressure was used for graft-recipient site dressing. The initial dressing was left undisturbed for 5 days, when the graft was uniformly pink, adherent and with a healthy capillary refill. Another 5 days of NPWT were added and the local treatment with zinc paste bandage (Varolast, Hartmann) was continued up to 2 weeks when the patient was allowed to resume walking. She stayed in the hospital for a total of 55 days and was discharged fully ambulatory. The patient considered the cosmetic appearance of his lower limb excellent (**Fig. 4**) and the reduction of the abdominal panniculus a bonus. In the past 3 years she did not experience any breakdown, nor did she develop contracture or suffered limitation of her range of motion. 

## Discussions

We chose to cover the wound with a full-thickness skin graft taken from the abdomen for several reasons, the main reason was to produce a minimum donor site morbidity, avoiding the lengthy secondary healing process of split thickens skin graft donor areas in a diabetic patient.

Beside the fact that a full-thickness skin graft will give a much better cosmetic and functional result, there is also a much subtle difference between thick and thin skin grafts in the way the dermal surface is processed, while the universal use of electric dermatome makes the harvest of a split skin graft an almost standardized procedure, with a sleek dermal face every time. The free hand defatting of a large thick graft with blades and scissors is less likely to achieve the same surface quality. This most certainly affects the early connection between graft and host vessels [**5**]. To overcome this drawback we used the VERSAJET Hydrosurgery System, which is a tangential cutting and suction instrument, which uses a high-speed jet stream of saline solution coupled with the Venturi effect [**6**]. Because all the power is generated at the tip of the hand piece, there is no effort on the part of the surgeon and, at least for large grafts, the defatting process exceeds in speed and quality the traditional methods.

The preparation of the graft-bed should be equally flawless. Compared with the classic cold knife degranulation technique, VERSAJET-powered tangential hydrodissection offers few remarkable advantages: in terms of ergonomics it allows the highest possible degree of flexibility and precision, it can effectively remove necrotic tissue from dermis, subcutaneous fat, muscle fascia or bone, being able to conform to any shape of the defect and generate a smooth transition between tissues of different resistance [**7**]. The wound bed can be thoroughly cleaned, by avoiding recontamination, preserving healthy tissue and minimizing blood loss.

A gauze-based negative pressure wound therapy system was used for the final graft-recipient site dressings. Every beneficial influence NPWT has on a regular wound, including an increasing perfusion, micro-deformation induced cellular proliferation and maintenance of wound homeostasis [**8**] is also beneficial for the graft take. For graft contouring and immobilization, fluid removal, insulation qualities and resistance to accidental deterioration NPWT is much better than any conventional dressing. 

## Conclusions

Based on the excellent result obtained in this patient, we think that the presented method is a good alternative to delay a full-thickness auto-grafting of cryopreserved avulsed skin [**9**] or to the use of Integra in combinations with meshed split-thickness skin grafts [**10**] in patients with redundant abdominal skin. VERSAJET could be a very useful tool in obtaining a full-thickness skin preparation from the degloved skin-envelope.

**Conflict of Interest**

None.

**Funding**

None.

**Ethical Approval:** Not required.
